# Cytometric cell-based assays for anti-striational antibodies in myasthenia gravis with myositis and/or myocarditis

**DOI:** 10.1038/s41598-019-41730-z

**Published:** 2019-03-27

**Authors:** Kenji Kufukihara, Yurika Watanabe, Takashi Inagaki, Koutaro Takamatsu, Shunya Nakane, Jin Nakahara, Yukio Ando, Shigeaki Suzuki

**Affiliations:** 1 Department of Neurology, Keio University School of Medicine, 35 Shinanomachi, Shinjuku-ku, Tokyo 160-8582 Japan; 2 Department of Neurology, Graduate School of Medical Sciences, Faculty of Life Sciences, Kumamoto University, 1-1-1 Honjo, Chuo-ku, Kumamoto 860-8556 Japan; 3 Department of Molecular Neurology and Therapeutics, Kumamoto University Hospital, 1-1-1 Honjo, Chuo-ku, Kumamoto 860-8556 Japan

## Abstract

The purposes of the present study were to identify anti-striational antibodies in myasthenia gravis (MG) patients with myositis and/or myocarditis using a combination of cell-based assays and flow cytometry (cytometric cell-based assays) and to describe the main clinical implications. Among 2,609 stored samples collected from all over Japan between 2003 and 2016, we had serum samples from 30 MG patients with myositis and/or myocarditis. Cytometric cell-based assays with titin, ryanodine receptor, and voltage-gated Kv1.4 were performed. Autoantibodies were determined by differences in phycoerythin fluorescence between the 293F cells and titin-transfected cells. MG patients with myositis and/or myocarditis as well as late-onset and thymoma-associated MG had anti-titin, anti-ryanodine receptor, and anti-Kv1.4 antibodies. In contrast, patients with early-onset MG, those with other myopathies and healthy controls did not have anti-titin or anti-Kv1.4 antibodies with some exceptions, but they possessed anti-ryanodine receptor antibodies. Thirty MG patients with myositis and/or myocarditis showed a severe generalized form, and 21 of them had thymoma. Anti-titin and anti-Kv1.4 antibodies were found in 28 (93%) and 15 (50%) patients, respectively, and all patients had at least one of these antibodies. Cytometric cell-based assays thus demonstrated that anti-striational antibodies are biomarkers of MG with myositis and/or myocarditis.

## Introduction

Anti-striational antibodies or anti-striated muscle antibodies were first described as serum immunoglobulins reacting with cross-striations of skeletal muscle in patients with myasthenia gravis (MG)^1^. Indirect immunofluorescence of animal skeletal muscle tissue was the original method for detecting anti-striational antibodies. Anti-striational antibodies were expected to serve as a predictive marker of thymoma in MG patients. However, immunoreactivity on indirect immunofluorescence was also observed in other disorders and normal controls. Because of the low specificity, the diagnostic utility of anti-striational antibodies was limited. Subsequent reports revealed the main autoantigens present in the skeletal and heart muscles, which included titin, ryanodine receptor, and voltage-gated potassium channel, Kv1.4^2–4^. The clinical significance of this finding is that these autoantibodies are frequently detected in MG patients with myositis and/or myocarditis throughout the entire MG disease course^5,6^.

Cell-based assays are now used in the detection of various autoantibodies against aquaporin-4, N-methyl-D-aspartate receptor and other neuronal and neuromuscular molecules^7–9^. Since cell-based assays are sensitive and specific for pathogenic extracellular epitopes, their clinical utility has been expanding. However, the disadvantage of cell-based assays is that the presence of autoantibodies is determined by visual evaluation using indirect immunofluorescence on culture cells. The process of discrimination between positive and negative results has plenty of room for improvement.

In this study, we identified anti-striational antibodies in MG patients with myositis and/or myocarditis using a combination of cell-based assays and flow cytometry (cytometric cell-based assays) and reported the main clinical implications.

## Results

### Patients and controls

**Figure 1:**
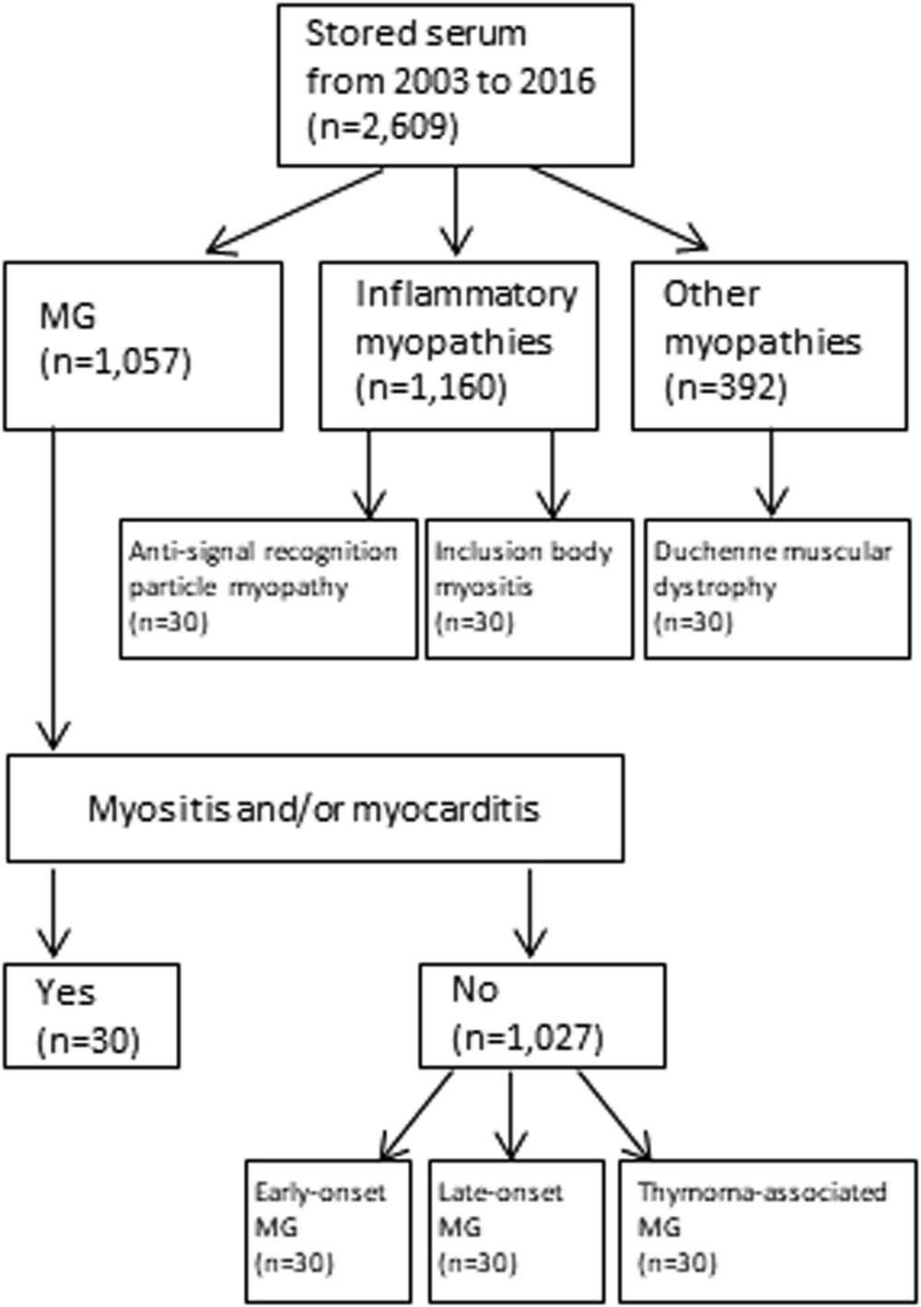
Study flow diagram. Patients and controls were indicated. MG = myasthenia gravis.

Between 2003 and 2017, we obtained serum from 2,609 patients with neuromuscular disorders for serological diagnosis from Keio MG clinics and other institutions across Japan. Stored serum samples were obtained from 1,057 patients with MG, 1,160 with inflammatory myopathies, and 392 patients with other myopathies (Fig. 1). Among 1,057 MG patients, we identified 30 patients who developed myositis and/or myocarditis during the clinical course. Nine of these patients were previously reported^6,10^. As the control groups, we randomly selected 30 patients with early-onset, 30 with late-onset, and 30 with thymoma-associated MG from the other 1,027 MG patients. In addition, we randomly selected 30 patients with anti-signal recognition particle myopathy, 30 with inclusion body myositis, and 30 with Duchenne muscular dystrophy as disease controls. Thirty healthy volunteers were also included.

### Autoantigens of cytometric cell-based assays

**Figure 2:**
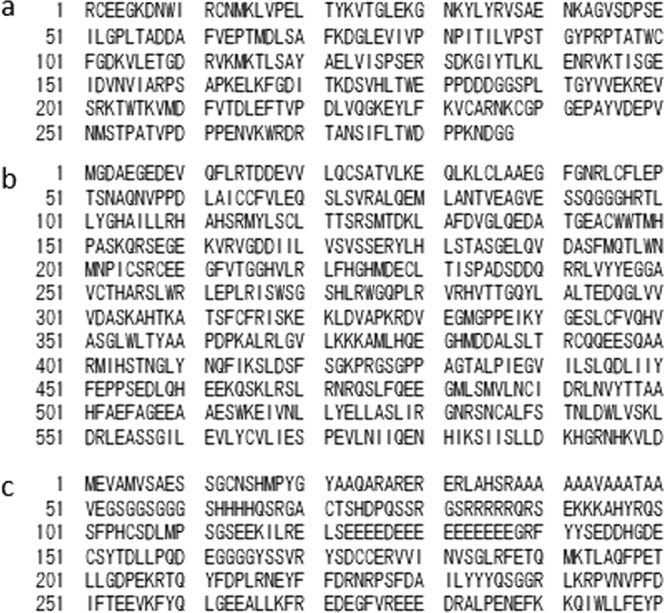
Amino acid sequences of autoantigens. (**a**) myasthenia gravis titin-30, (**b**) N-terminal residues of ryanodine receptor type 1, and (**c**) N-terminal residues of voltage-gated potassium channel Kv1.4.

We regarded three muscle proteins, titin, ryanodine receptor, and Kv1.4, as major autoantigens of anti-striational antibodies. We prepared the cDNA of three muscle antigens: MG titin-30 (accession number NM_X90568) (Fig. 2a), N-terminal residues of ryanodine receptor type 1 (accession number NM_00540) (Fig. 2b), and N-terminal residues of Kv1.4 (accession number NM_0022333) (Fig. 2c)^4,11,12^.

### Autoantibodies detection

We used the 293F cells and titin-transfected cells for autoantibodies detection. After excluding dead cells by 7-aminoactinomycin D (Thermo Fisher Scientific, Tokyo), the cells were assayed with a Gallios Flow Cytometer (Beckman Coulter, Brea, CA) and the data were analyzed with Kaluza software (Beckman Coulter, Brea, CA).

**Figure 3:**
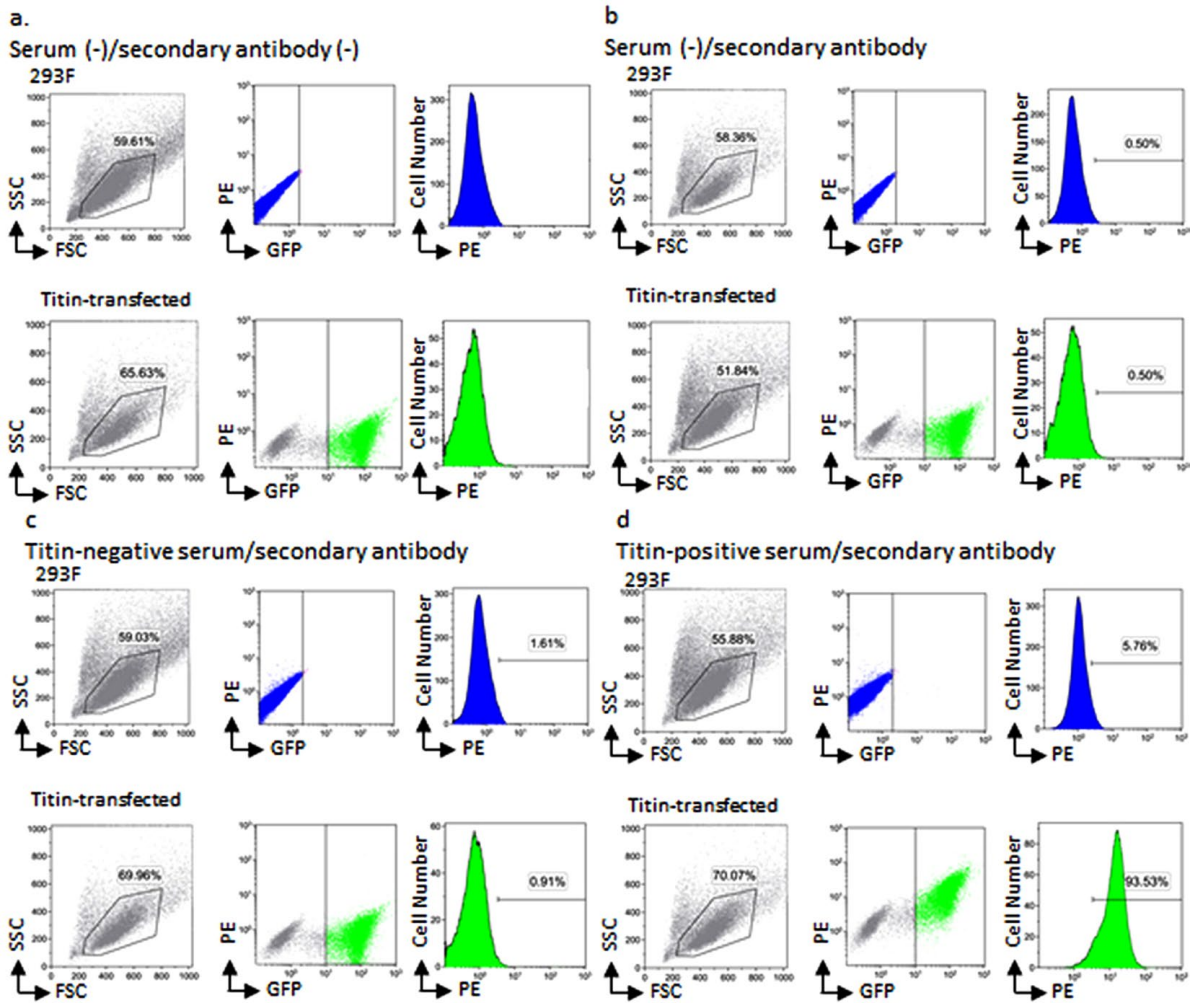
Flow cytometry of 293F cells and titin-transfected cells. (**a**) Gating strategy of 293F cells and titin-transfected cells. Cells were incubated without serum and secondary antibody. The gating strategy involved first identifying the 293F cell population by forward scatter (FSC) and side scatter (SSC) (left, upper). Live 293F cells were gated and measured fluorescent intensity of GFP and PE to define GFP-negative 293F cells (middle, upper). In addition, PE fluorescent intensity of GFP-negative 293 cells were analyzed by histogram plot (right, upper). Live titin-transfected cells were also gated by SSC versus FSC density plot (left, lower). We defined GFP-positive titin-transfected cells by PE versus GFP fluorescent intensity density plot (middle, lower). PE fluorescent intensity of titin-transfected cells were analyzed by histogram plot (right, lower). (**b**) Background PE fluorescent intensity was defined. Cells were incubated without serum and with secondary antibody. GFP-negative 293F cells and GFP-positive titin-transfected cells were gated. We defined background PE fluorescent intensity as shown in histogram plot (right, upper and right, lower). The proportion of background PE-positive cells was calculated as 0.5%. (**c**) The PE fluorescent intensity of titin-negative serum was analyzed. Cells were incubated with titin-negative serum and secondary antibody. The proportion of PE-positive/GFP-negative 293F cells and PE-positive/GFP-positive titin-transfected cells of titin-negative serum was calculated as 1.61% (right, upper) and 0.91% (right, lower), respectively. (**d**) The PE fluorescent intensity of titin-positive serum was analyzed. Cells were incubated with titin-positive serum and secondary antibody. The proportion of PE-positive/GFP-negative 293F cells and PE-positive/GFP-positive titin-transfected cells of titin-positive serum was calculated as 5.76% (right, upper) and 93.53% (right, lower), respectively. SSC = side scatter; PE = phycoerythrin; FSC = forward scatter, GFP = green fluorescent protein; PBS = phosphate-buffered saline.

Live 293F cells and titin-transfected cells were gated based on forward scatter (FSC) and side scatter (SSC) (Fig. 3a). With respect to titin-transfected cells, green fluorescent protein (GFP) positive cells were further separated from GFP negative cells. To obtain titin-transfected cells efficiently, we selected GFP-negative population in 293F cells and GFP-positive population in titin-transfected cells using different cut-off lines. Within the GFP positive cells, the fluorescence associated with phycoerythin (PE)-conjugated anti-human IgG secondary antibody was measured.

Autoantibody detection was based on the differences of the proportion of PE positive cells between the 293F cells and titin-transfected cells (Fig. 3b–d). To evaluate the background PE fluorescence of secondary antibodies, we incubated phosphate-buffered saline instead of human serum with culture cells. We defined background of PE fluorescence intensity as 0.5% of both culture cells (Fig. 3b). It was noted that 293F cells and titin-transfected cells had different background PE fluorescence intensity because of the existence of GFP molecule.

We also incubated MG serum with the 293F and titin-transfected cells and measured the proportion of PE positive cells of culture cells. Titin-negative serum, which was confirmed with enzyme-linked immunoassay (ELISA) (DLD Diagnostika GmbH, Hamburg, Germany), showed that the proportion of the PE positive cells was not increased in the titin-transfected cells (0.91%), compared to that in the 293F cells (1.61%) (Fig. 3c). In contrast, titin-positive serum indicated that the proportion of the PE positive cells was greater in the titin-transfected cells (93.53%) than in the 293F cells (5.76%) (Fig. 3d). Anti-ryanodine receptor and anti-Kv1.4 antibodies were also determined in the same procedures.

The antibody index was calculated as the proportion of cells expressing PE fluorescence in the titin-transfected cells divided by that in the 293F cells. With regard to titin-negative serum, the proportion of PE fluorescence in 293F cells was 1.61% and that in titin-transfected cells was 0.91%, resulting in an antibody index of 0.57. On the other hand, the antibody index of titin-positive serum was 16.2. In contrast, when we selected GFP-negative population in 293F cells and GFP-positive population in titin-transfected cells using same cut-off lines (Supplementary File, Data 1), the antibody index of titin-negative and titin-positive serum were 0.58 and 17.2, respectively.

**Figure 4:**
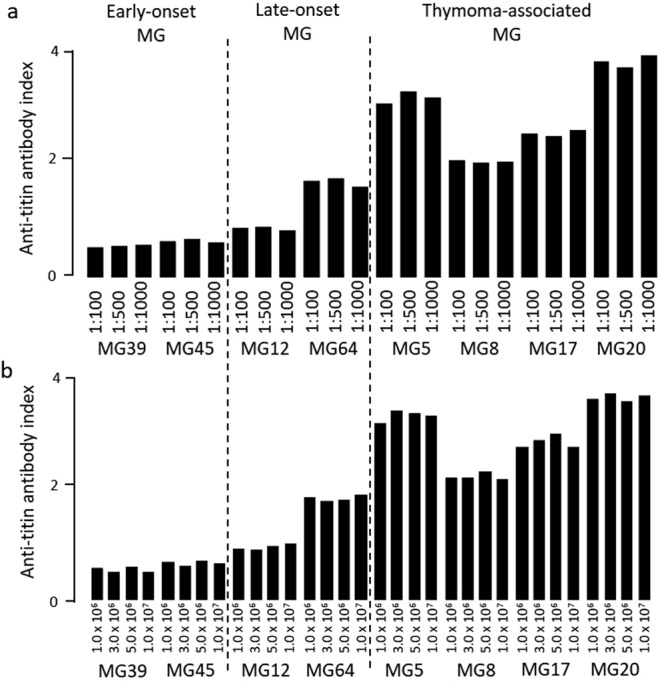
Anti-titin antibody index. (**a**) different serum dilutions and (**b**) different cell numbers of titin-transfected cells.

We studied the differences in the antibody index among different dilutions of serum and different numbers of culture cells. Representative findings of thymoma-associated MG patients are indicated in Fig. 4. Experiments were conducted in triplicate. We confirmed that same results were obtained using different dilutions of patients’ serum (1:100, 1:500, and 1:1000) (Fig. 4a). In addition, there were no differences in the antibody index among the cultures containing 1.0 × 10^6^, 3.0 × 10^6^, 5.0 × 10^6^, and 1.0 × 10^7^ cells (Fig. 4b). Similarly, these findings were observed in patients with late-onset or early-onset MG.

### Distribution of antibody index

**Figure 5:**
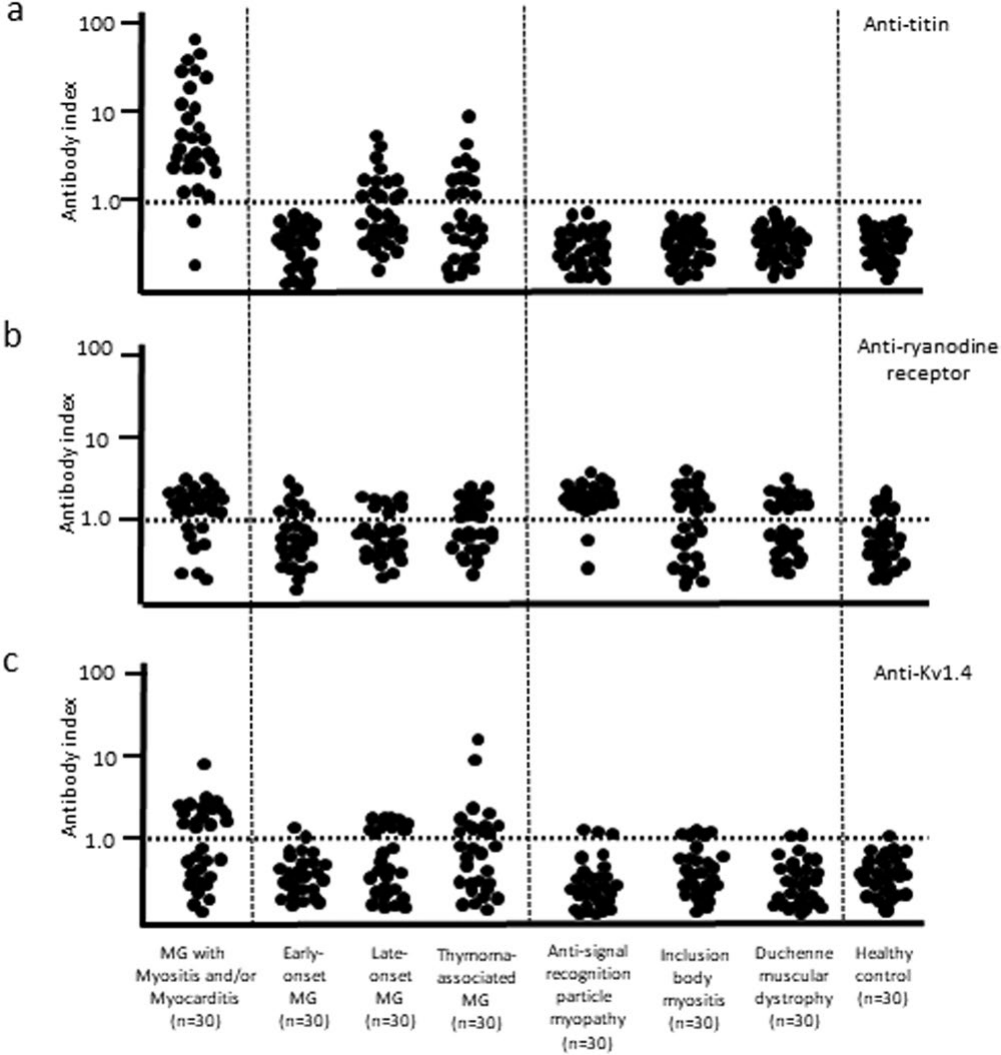
Distribution of antibody index of anti-striational antibodies. (**a**) anti-titin antibodies, (**b**) anti-ryanodine receptor antibodies, and (**c**) anti-Kv1.4 antibodies. The dotted line denotes the cut-off level between positive and negative results. MG = myasthenia gravis.

The distribution of the antibody index is shown in Fig. 5. Theoretically, if immunoglobulins in titin-positive serum bound to the titin-transfected cells more than to the 293F cells, the antibody index was calculated to be over 1.0. Thus, we determined the cut-off antibody index between positive and negative results to be 1.0. All results are available in Supplementary File (Data 2–4). Anti-titin antibodies were detected in 93% (28/30) of MG patients with myositis and/or myocarditis (Fig. 5a). With regard to other MG patients, 43% (13/30) of those with late-onset and 40% (12/30) of those with thymoma-associated, but none of those with early-onset MG had anti-titin antibodies. However, among the disease controls and healthy controls, all of them lacked anti-titin antibodies.

Anti-ryanodine receptor antibodies were detected in 73% (22/30) of MG patients with myositis and/or myocarditis and in 41% (37/90) of the other MG patients (Fig. 5b). Unexpectedly, 61% (55/90) of the disease control patients and 23% (7/30) of healthy controls showed positivity for anti-ryanodine receptor antibodies. In contrast, anti-Kv1.4 antibodies were found in 50% (15/30) of MG patients with myositis and/or myocarditis (Fig. 5c). Anti-Kv1.4 antibodies were also detected in 28% (25/90) of the other MG patients and were especially prominent in late-onset and thymoma-associated MG patients. Seropositive results were seen in 10% (9/90) of disease control patients and 3% (1/30) of healthy controls.

MG patients in the control groups (early-onset, late-onset and thymoma-associated) were all seropositive for anti-acetylcholine receptor antibodies. Additionally, we evaluated three early-onset MG patients with anti-muscle-specific kinase antibodies. They were all negative for anti-titin, anti-ryanodine receptor and anti-Kv1.4 antibodies.

### Clinical features

The clinical features of the 30 MG patients with myositis and/or myocarditis are shown in Table 1. They were eight men and 22 women. The mean age was 60 ± 12 years (range 34–82 years). It is noted that the mean age was evaluated by the age of development of myositis and/or myocarditis. However, the mean age of MG patients in the control groups was defined as the onset age of MG. Disease subtypes included 21 patients with thymoma-associated, eight with late-onset, and one with early-onset MG. All patients had generalized MG with severe disease involvement. The MG Foundation of America classification showed 17 patients in class 5, three in class 4, and 10 in class 3. Serum creatine kinase levels were elevated with an average of 3,466 ± 6,020 IU/L (range 43–27,170 IU/L). All MG patients had anti-acetylcholine receptor antibodies except one, who was also negative for anti-muscle-specific kinase antibodies.

**Table 1:** Characteristics of 30 MG patients with myositis and/or myocarditis.

Patient/Age (y)/sex	Type	MGFA Class	Creatine kinase (IU/L)	Anti- AChR (nM)	Myositis/myocarditis	Outcome	Anti-titin	Anti-Kv1.4	Former anti- titin	Former anti- Kv1.4
1/34/F	TMG	5	27,170	240	+/+	Dead	+	+	+	+
2/62/M^a,b^	TMG	5	9,835	280	+/+	Dead	+	+	+	+
3/69/F^b^	TMG	5	1,250	6.5	+/+	Dead	+	+	+	+
4/80/F^b^	TMG	5	3,107	344	+/+	Alive	+	−	+	+
5/52/F	TMG	3b	1,270	28	+/+	Dead	+	+	+	+
6/66/F	LOMG	3a	16,489	<0.2	+/+	Alive	−	+	−	+
7/48/F^a^	EOMG	5	3,482	92	+/−	Alive	+	−	+	+
8/50/F	TMG	5	99	800	+/−	Alive	+	+	+	+
9/62/F^a^	LOMG	5	3,193	590	+/−	Alive	+	−	+	−
10/63/F	TMG	5	3,432	19	+/−	Alive	+	+	+	+
11/73/F	TMG	5	4,424	70	+/−	Alive	+	−	+	+
12/80/M	LOMG	5	2,300	99	+/−	Alive	+	−	+	ND
13/68/F^a^	LOMG	4b	362	150	+/−	Alive	+	−	+	+
14/49/F	TMG	3b	465	32	+/−	Alive	+	−	+	−
15/58/F	TMG	3b	11,117	87	+/−	Alive	+	+	+	+
16/63/F	LOMG	3b	965	112	+/−	Alive	+	+	+	+
17/59/M	TMG	3a	10,226	12	+/−	Alive	+	+	+	+
18/45/F^a,b^	TMG	5	67	390	−/+	Alive	+	−	−	+
19/46/M	TMG	5	267	86	−/+	Dead	+	−	+	ND
20/52/F	TMG	5	810	230	−/+	Dead	+	+	+	+
21/53/F^a,b^	TMG	5	397	130	−/+	Alive	+	−	−	+
22/59/M	TMG	5	102	23	−/+	Alive	+	−	−	−
23/76/M	TMG	5	745	2.5	−/+	Dead	+	−	−	ND
24/82/F	LOMG	5	401	48	−/+	Alive	−	+	−	+
25/51/M^b^	TMG	4b	66	57	−/+	Alive	+	+	−	+
26/74/M	LOMG	4a	120	72	−/+	Alive	+	−	−	ND
27/59/F	TMG	3b	681	180	−/+	Dead	+	+	+	+
28/60/F	TMG	3b	69	200	−/+	Alive	+	−	+	ND
29/63/F	TMG	3b	43	370	−/+	Alive	+	+	−	+
30/57/F	LOMG	3a	1,013	15	−/+	Alive	+	−	+	ND

Abbreviations: AChR = acetylcholine receptor; EOMG = early-onset MG; LOMG = late-onset MG; ND = no data; MG = myasthenia gravis; MGFA = MG Foundation of America; TMG = thymoma-associated MG.

^a^Patients previously reported in ref.^6^.

^b^Patients previously reported in ref.^10^.

Six (patients 1–6) developed both myositis and myocarditis, 11 (patients 7–17) developed myositis alone, and 13 (patients 18–30) developed myocarditis alone. All patients received immunomodulatory therapy such as corticosteroids, tacrolimus and cyclosporin microemulsion. Acute exacerbation of symptoms of myositis and/or myocarditis required the plasmapheresis or intravenous immunoglobulin therapy. The clinical features were severe, but the responses to immunomodulatory therapy were generally favorable. Clinical outcomes revealed that eight patients died of severe myocarditis despite the use of intensive immunosuppressive agents and supportive care. There were no deaths due to myasthenic crisis.

Cytometric cell-based assays revealed that 13 MG patients with myositis and/or myocarditis had both anti-titin and anti-Kv1.4 antibodies. Two patients who did not have anti-titin antibodies were positive for anti-Kv1.4 antibodies. We compared the results of the cytometric cell-based assay to those of the former detection methods. Anti-titin antibodies and anti-Kv1.4 antibodies were measured using a commercially available ELISA and a protein immunoprecipitation assay using isotope-labeled RD cells, respectively^4,11^. Positivity for anti-titin antibodies was increased from 21 patients with the former method to 28 patients with the cytometric cell-based assay. In contrast, the seropositive results of anti-Kv1.4 antibodies were decreased from 21 patients with the former method to 15 patents with the cytometric cell-based assay.

**Figure 6:**
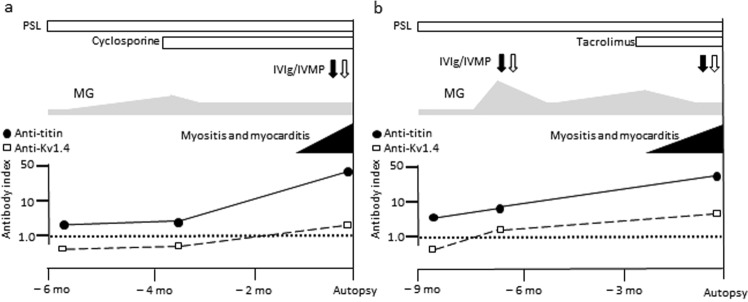
Clinical courses of patients with myasthenia gravis with myositis and myocarditis. (**a**) patient 1 and (**b**) patient 2. PSL = prednisolone; IVIg = intravenous immunoglobulin; IVMP = intravenous methylprednisolone.

Representative cases (patient 1 and patient 2 in Table 1) suffering from severe myositis and myocarditis are shown (Fig. 6a,b). They had invasive thymoma at an advanced stage. MG was treated with prednisolone and immunosuppressive agents. Myositis and myocarditis suddenly developed at approximately 4 years and 16 years after the onset of MG, respectively. Both patients were cared for in intensive care units and received additional treatment including intravenous immunoglobulin and methylprednisolone. However, they suffered from heart failure and recurrent ventricular tachycardia and died of myocarditis.

**Figure 7:**
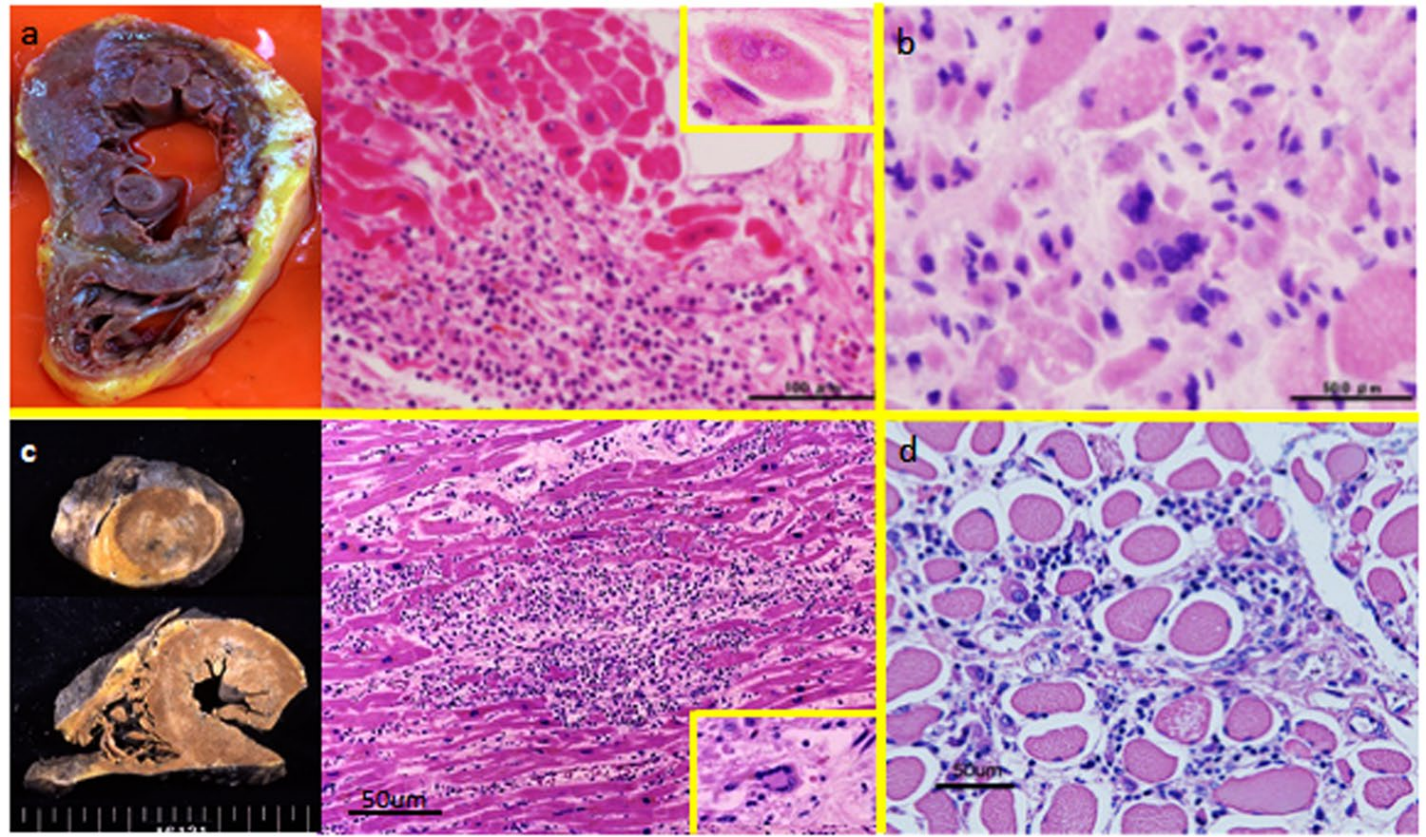
Autopsy findings. (**a**) myocarditis and (**b**) myositis of patient 1, and (**c**) myocarditis and (**d**) myositis of patient 2. Patient 2 was previously reported in ref.^6^.

Autopsy findings showed massive necrosis of the myocardium (Fig. 7a,c). There were many infiltrated lymphocytes including giant cells in the heart muscles. Diffuse cellular infiltration and severe necrosis of skeletal muscles were also observed (Fig. 7b,d). Serial changes in the antibody index showed that anti-titin antibodies were present before the onset of myositis and myocarditis. In contrast, it is likely that the appearance of anti-Kv1.4 antibodies was correlated with the development of myositis and myocarditis.

## Discussion

The results of the present study can be summarized as follows: (i) we established cytometric cell-based assays for anti-striational antibodies; (ii) anti-titin and anti-Kv1.4, but not anti-ryanodine receptor antibodies, were preferentially detected in patients with MG with myositis and/or myocarditis, and those with late-onset and thymoma-associated MG, but not in patients with early-onset MG or other myopathies or in healthy controls; (iii) MG with myositis and/or myocarditis was characterized by female dominancy, the presence of thymoma, severe neuromuscular symptoms and lethal outcome; and (iv) all patients with MG with myositis and/or myocarditis were seropositive for anti-titin or anti-Kv1.4 antibodies.

Among anti-striational antibodies, many investigators have studied the clinical significance of autoantibodies to titin, which is the most abundant intracellular muscle protein and has a huge molecular size^13^. The epitope recognized by autoantibodies, which was determined to be MG titin-30, was common between the prior enzyme-linked immunosorbent assay and current cytometric cell-based assays^11^. We showed an improvement in seropositivity in MG patients with myositis and/or myocarditis detected by cytometric cell-based assay. This finding suggests that cytometric cell-based assay is a sensitive method for detecting anti-titin antibodies without false positive results. In the same manner, Stergiou *et al*. also developed a very sensitive radioimmunoprecipitation assay for anti-titin antibodies and proposed that this method was a useful tool for the diagnosis of seronegative MG patients^14^.

Ryanodine receptors and voltage-gated potassium channel Kv1.4 are membrane proteins of skeletal and heart muscles. The presence of anti-ryanodine receptor and anti-Kv1.4 antibodies in patients with MG has been successfully established. However, anti-ryanodine receptor antibodies were detected in patients with other myopathies and in healthy controls. We speculate that the lack of sensitivity in the indirect immunofluorescence assays for anti-striational antibodies may be related to the false positive results of anti-ryanodine receptor antibodies. Anti-ryanodine receptor antibodies are involved in the disturbance of excitation-contraction coupling in skeletal muscle tissue^15^. On the other hand, our results suggest that the production of anti-ryanodine receptor antibodies may be a secondary phenomenon related to the muscle necrosis observed in immune-mediated necrotizing myopathy or muscular dystrophy.

In contrast, the false positivity of anti-Kv1.4 antibodies may be within the allowable range. However, six MG patients with myositis and/or myocarditis showed different results for anti-Kv1.4 antibodies between the prior and current methods. The original method for detecting anti-Kv1.4 antibodies was the protein immunoprecipitation assay using isotope-labeled RD cells^4^. In this assay, 70-kDa autoantigens in the electrophoresis were regarded as voltage-gated potassium channel Kv1.4. Other molecules associated with potassium channels with similar molecular weights could be potentially regarded as Kv1.4 in the prior assay.

The strength of cytometric cell-based assays is that the results depend on the PE fluorescence measured by flow cytometry. It is possible to exclude investigators’ bias when evaluating immunoreactivity on indirect immunofluorescence. We believe the cytometric cell-based assay for anti-titin and anti-Kv1.4 antibodies is a valuable tool for detecting anti-striational antibodies. In addition, we emphasize that all MG patients with myositis and/or myocarditis had anti-titin or anti-Kv1.4 antibodies. Thus, anti-striational antibodies are good serological markers for lethal events during the follow-up of MG patients.

Among many of the autoimmune diseases observed in MG patients, myositis and/or myocarditis are the most critical disorders, although the frequency of these in all MG patients is generally low, ranging from 0.9% to 2.3%^6,16,17^. Our clinical analysis revealed that MG patients with thymoma, female patients, and older patients were prone to developing myositis and/or myocarditis. Since MG was generally severe in these patients, it is necessary to discriminate between an MG crisis and respiratory insufficiency due to myocarditis. We emphasize that myocarditis is a critical disease in MG patients because the mortality due to myocarditis in our cohort of MG patients was 42%. Severe heart failure and lethal arrythmias were resistant to immunotherapy and supportive care. In this regard, it is worth noting a previous report in which the sudden death of thymoma-associated MG patients was suggested to be due to myocarditis^18^.

Recently, the clinical significance of anti-striational antibodies has been reconsidered. Immune checkpoint inhibitors are causative agents in the development of new onset MG. Immune checkpoint inhibitors-related MG is usually severe and is frequently accompanied by myositis and/or myocarditis^19^. Anti-striational antibodies are detectable in the serum of these patients and are expected to be serological markers for serious immune-related adverse events. In fact, the clinical guidelines of the American Society of Clinical Oncology recommend the detection of anti-striational antibodies for the diagnosis of MG during the treatment of immune checkpoint inhibitors^20^.

There are three limitations in the present study. First, we did not study the prevalence of the development of myositis and/or myocarditis in MG patients having anti-striational antibodies. It is noted that not all MG patients with anti-striational antibodies suffer from myositis and/or myocarditis. Second, MG subsets are usually divided into early-onset, late-onset and thymoma-associated. Based on the present and our previous studies, we consider that late-onset MG and thymoma-associated MG may have common immunological aspects in the presence of anti-striational antibodies and a weak correlation to human leucocyte antigen^21^. However, we had the other specific subset of MG patients with myositis and/or myocarditis. It was noted that this subset was defined by different pathophysiological events. Third, we did not evaluate the possibility of other epitopes of anti-ryanodine receptor antibodies. Ryanodine receptor, a calcium release channel located in the sarcoplasmic reticulum, has skeletal and cardiac forms. It is a protein containing 5,035 amino acids with a molecular weight of 565-kDa. Anti-ryanodine receptor antibodies were first identified using western blot for the presence of antibodies to the protein of the sarcoplasmic reticulum from rabbit skeletal muscle^3^. In addition, several epitopes in both the N- and C- terminus of ryanodine receptor sequence were identified and used as antigenic peptide in ELISA^12,22,23^. Unfortunately, since there were no standard methods, we could not perform validation of detection methods for anti-ryanodine receptor antibodies. A future study to determine the pathogenicity of anti-striational antibodies and to confirm their potential utility as a biomarker with a large series of patients is necessary.

In conclusion, cytometric cell-based assays for anti-striational antibodies are sensitive biomarkers of MG with myositis and/or myocarditis.

## Methods

### Clinical samples and diagnosis

Serum samples were stored at −30 °C and treated in accordance with current legislation approved by the Institutional Review Boards of Keio University (no. 20090278). All of the patients gave written informed consent.

The diagnosis of MG was made based on the patient exhibiting the typical history and signs of fluctuating weakness in the voluntary muscles, the presence of serum anti-acetylcholine receptor or anti-muscle-specific kinase antibody, and abnormal findings of neuromuscular junction transmission^24^. MG subtypes were divided into early-onset, late-onset, and thymoma-associated MG using a cut-off age of 50 years. MG severity was evaluated according to the MG Foundation of America system^25^. The diagnosis of myositis was based on clinical symptoms, serum creatine kinase levels, electromyography, muscle MRI, and histology. The diagnosis of myocarditis was based on cardiac symptoms that had no other causes, laboratory findings including abnormalities in electrocardiography and echocardiography, and histological evaluation. Coronary artery diseases were excluded by angiography.

### Cytometric cell-based assays

The cDNAs of titin, ryanodine receptor, and Kv1.4 were subcloned into mammalian expression vectors containing GFP (ATUM, Newark, CA). Human embryonic kidney 293F cells (Thermo Fisher Scientific, Tokyo) were utilized in transfection with these vectors. The 293F and transfected cells were cultured in Dulbecco’s Modified Eagle Medium with 4.5 g/L D-glucose, L-glutamine supplemented with 10% fetal bovine serum, 1% penicillin and streptomycin. The stable transfected cells were taken from the culture flask with 0.05% ethylenediaminetetraacetic acid/phosphate-buffered saline.

To test for the presence of antibodies, 5.0 × 10^6^ culture cells were incubated on ice for 1 hour with the patients’ serum (1:100). Then the cells were washed and incubated with PE-labeled secondary anti-human IgG (1:1,000) antibodies (Thermo Fisher Scientific, Tokyo) for 30 minutes. After incubating with 7-aminoactinomycin D (Thermo Fisher Scientific, Tokyo), the cells were applied to a flow cytometer and analyzed with Kaluza software (Gallios, Beckman Coulter, Brea, CA).

### Autoantibodies detection

Serum anti-acetylcholine receptor and anti-muscle-specific kinase antibodies were measured by conventional radio radioimmunoassays. The main immunogenic region of titin is identified as MG titin-30 near the A/I-band junction, although titin is a giant protein (3,000-kDa) abundantly in the skeletal and cardiac sarcomere. Autoantibodies to titin are widely determined by the standard method using a commercially available ELISA. We evaluated anti-titin antibodies using the ELISA system (DLD Diagnostika GmbH, Hamburg, Germany)^11^. Anti-Kv1.4 antibodies were identified as 70-kDa autoantigens in a protein immunoprecipitation assay using isotope-labeled RD cells^4^.

### Statistical methods

All data are presented as means ± standard deviation. Statistical analyses were performed using SPSS (version 10). All analyses were performed using statistical analysis software (IBM/SPSS version 20, Armonk, NY).

## Supplementary information

Dataset 1, 2, 3, 4

## Data Availability

The datasets generated and analyzed during the current study are available from the corresponding author on reasonable request.
