# Supplementary material for: Cytometric cell-based assays for anti-striational antibodies in myasthenia gravis with myositis and/or myocarditis
Source: Sci Rep. 2019 Mar 27;9:5284. doi: 10.1038/s41598-019-41730-z (PMC6437199; doi:10.1038/s41598-019-41730-z)
Supplement: Supplementary file 1 — Dataset 1, 2, 3, 4 [file 41598_2019_41730_MOESM1_ESM.docx]

**Supplemental data**

**Cytometric cell-based assays for anti-striational antibodies in myasthenia gravis (MG) with myositis and/or myocarditis**

Kenji Kufukihara^1^, Yurika Watanabe^1^, Takashi Inagaki^1^, Koutaro Takamatsu^2^, Shunya Nakane^2,3^, Jin Nakahara^1^, Yukio Ando^2^, Shigeaki Suzuki^1*^

^1^Department of Neurology, Keio University School of Medicine, 35 Shinanomachi, Shinjuku-ku, Tokyo 160-8582, Japan

^2^Department of Neurology, Graduate School of Medical Sciences, Faculty of Life Sciences, Kumamoto University, 1-1-1 Honjo, Chuo-ku, Kumamoto 860-8556, Japan

^3^Department of Molecular Neurology and Therapeutics, Kumamoto University Hospital, 1-1-1 Honjo, Chuo-ku, Kumamoto 860-8556, Japan

Supplementary data 1

Flow cytometry of 293F cells and titin-transfected cells. When we selected GFP-negative population in 293F cells and GFP-positive population in titin-transfected cells using different cut-off lines (Fig. 3), the antibody index of titin-negative and titin-positive serum were 0.57 and 16.2, respectively. In contrast, when we selected GFP-negative population in 293F cells and GFP-positive population in titin-transfected cells using same cut-off lines (supplementary data 1), the antibody index of titin-negative and titin-positive serum were 0.58 and 17.2, respectively.


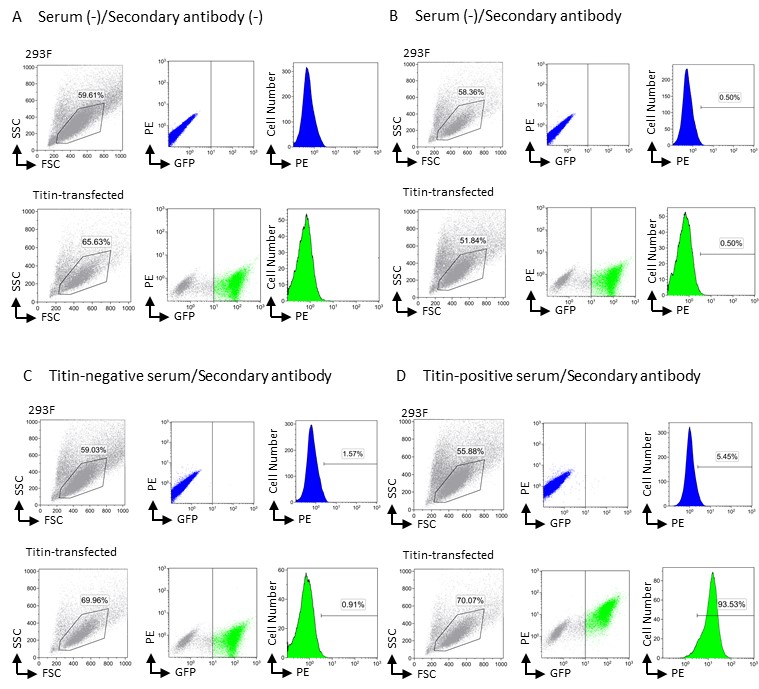


Supplementary data 2

Anti-titin antibody index

| Titin-transfected cells | Cell number | Proportion of PE positive 293F cells | Proportion of PE positive titin-transfected cells | Antibody index |
| --- | --- | --- | --- | --- |
| MG with Myositis and/or Myocarditis |  |  |  |  |
| 1 | 44,658 | 0.41 | 2.76 | 6.73 |
| 2 | 44,845 | 1.33 | 8.77 | 6.59 |
| 3 | 42,892 | 44.46 | 62.95 | 1.41 |
| 4 | 43,622 | 1.95 | 16.18 | 8.29 |
| 5 | 44,631 | 0.86 | 9.65 | 11.22 |
| 6 | 44,401 | 21.95 | 5.41 | 0.24 |
| 7 | 40,921 | 11.18 | 39.52 | 3.53 |
| 8 | 44,231 | 0.76 | 4.69 | 6.17 |
| 9 | 27,952 | 53.35 | 80.69 | 1.51 |
| 10 | 42,430 | 13.3 | 74.05 | 5.56 |
| 11 | 43,883 | 3.86 | 63.19 | 16.37 |
| 12 | 45,337 | 0.89 | 19.3 | 21.68 |
| 13 | 44,606 | 0.47 | 2.3 | 4.89 |
| 14 | 44,362 | 20.32 | 76.84 | 3.78 |
| 15 | 44,215 | 7.62 | 85.22 | 11.18 |
| 16 | 44,042 | 15 | 60.74 | 4.04 |
| 17 | 44,652 | 0.65 | 1.06 | 1.63 |
| 18 | 44,490 | 2.23 | 10.44 | 4.68 |
| 19 | 45,951 | 1.58 | 56.04 | 35.46 |
| 20 | 44,435 | 3.07 | 5.03 | 1.63 |
| 21 | 45,810 | 2.1 | 48.02 | 22.86 |
| 22 | 41,566 | 0.43 | 30.85 | 71.74 |
| 23 | 44,763 | 0.99 | 39.85 | 40.25 |
| 24 | 44,002 | 1.77 | 1.15 | 0.64 |
| 25 | 47,513 | 6.76 | 44.19 | 6.53 |
| 26 | 46,393 | 13.29 | 45.12 | 3.39 |
| 27 | 47,544 | 1.27 | 55.13 | 43.4 |
| 28 | 46,488 | 0.46 | 0.63 | 1.36 |
| 29 | 42,349 | 14.7 | 65.02 | 4.42 |
| 30 | 45,752 | 0.65 | 2.38 | 3.66 |
| Early-onset MG |  |  |  |  |
| 1 | 44,254 | 1.38 | 0.88 | 0.63 |
| 2 | 44,143 | 24.66 | 2.73 | 0.11 |
| 3 | 43,530 | 10.77 | 1.59 | 0.14 |
| 4 | 24,072 | 6.32 | 2.1 | 0.33 |
| 5 | 35,309 | 1.05 | 0.64 | 0.6 |
| 6 | 23,454 | 0.86 | 0.45 | 0.52 |
| 7 | 44,052 | 1.03 | 0.76 | 0.73 |
| 8 | 35,553 | 2.8 | 0.5 | 0.17 |
| 9 | 27,713 | 0.94 | 0.59 | 0.62 |
| 10 | 44,688 | 1.04 | 0.78 | 0.75 |
| 11 | 37,835 | 4.12 | 0.66 | 0.16 |
| 12 | 35,281 | 0.86 | 0.52 | 0.6 |
| 13 | 38,060 | 1.13 | 0.63 | 0.55 |
| 14 | 30,064 | 11.82 | 1.56 | 0.13 |
| 15 | 34,279 | 0.92 | 0.57 | 0.61 |
| 16 | 30,901 | 7.63 | 3.16 | 0.41 |
| 17 | 23,800 | 1.02 | 0.55 | 0.53 |
| 18 | 40,850 | 1.58 | 0.85 | 0.53 |
| 19 | 19,045 | 21.2 | 1.96 | 0.09 |
| 20 | 27,018 | 2.52 | 0.67 | 0.26 |
| 21 | 19,086 | 3.83 | 3.03 | 0.79 |
| 22 | 44,114 | 1.1 | 0.74 | 0.67 |
| 23 | 28,792 | 11.6 | 2.33 | 0.2 |
| 24 | 43,797 | 1.63 | 0.86 | 0.52 |
| 25 | 22,718 | 12.23 | 1.85 | 0.15 |
| 26 | 31,925 | 4.71 | 1.09 | 0.23 |
| 27 | 23,759 | 2.19 | 0.89 | 0.4 |
| 28 | 32,081 | 31.24 | 10.38 | 0.33 |
| 29 | 31,212 | 1.13 | 0.84 | 0.74 |
| 30 | 43,467 | 2.02 | 0.97 | 0.48 |
| Late-onset MG |  |  |  |  |
| 1 | 36,670 | 1.51 | 0.82 | 0.54 |
| 2 | 31,738 | 36.17 | 95.72 | 2.64 |
| 3 | 35,172 | 0.77 | 0.76 | 0.98 |
| 4 | 36,308 | 17.88 | 48.36 | 2.7 |
| 5 | 33,564 | 43.83 | 57.73 | 1.31 |
| 6 | 34,270 | 1.4 | 0.79 | 0.56 |
| 7 | 33,104 | 1.41 | 1.14 | 0.8 |
| 8 | 33,322 | 1.24 | 0.77 | 0.62 |
| 9 | 33,796 | 2.25 | 1.16 | 0.51 |
| 10 | 34,605 | 4.29 | 31.05 | 7.23 |
| 11 | 34,968 | 1.71 | 1.01 | 0.59 |
| 12 | 34,947 | 1.14 | 1.05 | 0.92 |
| 13 | 33,684 | 1.89 | 1.16 | 0.61 |
| 14 | 34,990 | 1.82 | 1.51 | 0.82 |
| 15 | 40,959 | 28.92 | 1.12 | 0.03 |
| 16 | 34,432 | 2.69 | 1.14 | 0.42 |
| 17 | 46,609 | 21.61 | 74.29 | 3.43 |
| 18 | 34,539 | 1.52 | 1.37 | 0.9 |
| 19 | 34,435 | 1.68 | 1.41 | 0.83 |
| 20 | 34,857 | 0.83 | 0.84 | 1.01 |
| 21 | 31,866 | 1.33 | 1.08 | 0.81 |
| 22 | 35,079 | 0.77 | 0.93 | 1.2 |
| 23 | 35,618 | 0.84 | 0.91 | 1.08 |
| 24 | 35,635 | 3.32 | 26.28 | 7.91 |
| 25 | 35,211 | 0.97 | 3.18 | 3.27 |
| 26 | 33,272 | 0.87 | 1.09 | 1.25 |
| 27 | 34,247 | 3.12 | 8.02 | 2.57 |
| 28 | 35,765 | 1.01 | 0.78 | 0.77 |
| 29 | 35,038 | 1.08 | 1.16 | 1.07 |
| 30 | 33,602 | 28.9 | 20.43 | 0.7 |
| Thymoma-associated MG |  |  |  |  |
| 1 | 51,241 | 0.92 | 9.33 | 10.14 |
| 2 | 15,902 | 80.03 | 97 | 1.21 |
| 3 | 16,925 | 10.08 | 2.28 | 0.23 |
| 4 | 12,062 | 6.58 | 59.85 | 9.09 |
| 5 | 42,319 | 26.23 | 91.14 | 3.47 |
| 6 | 42,966 | 45.87 | 2.47 | 0.01 |
| 7 | 27,225 | 7.49 | 1.75 | 0.23 |
| 8 | 15,504 | 89.63 | 7.07 | 0.08 |
| 9 | 42,774 | 43.78 | 9.48 | 0.21 |
| 10 | 19,802 | 8.13 | 4.08 | 0.5 |
| 11 | 9,950 | 8.58 | 92.1 | 10.73 |
| 12 | 26,779 | 26.62 | 2.31 | 0.09 |
| 13 | 13,045 | 1.68 | 5.22 | 3.1 |
| 14 | 43,552 | 83.73 | 4.62 | 0.06 |
| 15 | 44,068 | 81.54 | 6.52 | 0.08 |
| 16 | 44,788 | 1.45 | 6.92 | 4.77 |
| 17 | 45,121 | 1.78 | 12.27 | 6.89 |
| 18 | 44,169 | 6.91 | 1.8 | 0.26 |
| 19 | 43,971 | 2.07 | 7.77 | 3.75 |
| 20 | 44,380 | 19.92 | 19.83 | 0.99 |
| 21 | 43,966 | 1.51 | 6.63 | 4.39 |
| 22 | 43,691 | 61.74 | 2.05 | 0.03 |
| 23 | 43,921 | 41,25 | 1.91 | 0.05 |
| 24 | 16,755 | 31.34 | 29.7 | 0.94 |
| 25 | 44,508 | 15.94 | 7.03 | 0.44 |
| 26 | 43,565 | 1.62 | 6.95 | 4.29 |
| 27 | 41,941 | 48.57 | 3.92 | 0.08 |
| 28 | 39,596 | 0.56 | 0.36 | 0.64 |
| 29 | 42,851 | 1.85 | 6.77 | 3.65 |
| 30 | 43,625 | 7.28 | 2.31 | 0.32 |
| Anti-signal recognition particle myopathy |  |  |  |  |
| 1 | 40,123 | 3.67 | 0.96 | 0.26 |
| 2 | 41,009 | 2.91 | 1.13 | 0.38 |
| 3 | 53,746 | 0.59 | 0.13 | 0.22 |
| 4 | 45,369 | 2.51 | 0.96 | 0.38 |
| 5 | 53682 | 0.87 | 0.19 | 0.21 |
| 6 | 40,816 | 0.77 | 0.49 | 0.63 |
| 7 | 40,797 | 2.74 | 1.22 | 0.44 |
| 8 | 40,399 | 5.37 | 2.25 | 0.41 |
| 9 | 41,358 | 39.23 | 21.19 | 0.54 |
| 10 | 53,073 | 0.91 | 0.17 | 0.18 |
| 11 | 43,207 | 9.8 | 1.48 | 0.15 |
| 12 | 40,709 | 12.19 | 5.32 | 0.43 |
| 13 | 43,648 | 0.96 | 0.52 | 0.54 |
| 14 | 42,961 | 9.76 | 1.91 | 0.19 |
| 15 | 43,354 | 19.29 | 3.14 | 0.16 |
| 16 | 43,061 | 3.96 | 0.76 | 0.19 |
| 17 | 42,116 | 71.88 | 44.34 | 0.61 |
| 18 | 44,003 | 0.78 | 0.51 | 0.65 |
| 19 | 43,856 | 6.76 | 1.44 | 0.21 |
| 20 | 42,809 | 12.35 | 1.97 | 0.15 |
| 21 | 41,989 | 13.08 | 2.38 | 0.18 |
| 22 | 43,627 | 10.53 | 2.08 | 0.19 |
| 23 | 43,557 | 6.01 | 1.86 | 0.3 |
| 24 | 42,708 | 2.15 | 0.65 | 0.3 |
| 25 | 43,756 | 20.37 | 5.89 | 0.28 |
| 26 | 43,013 | 10.24 | 2.53 | 0.24 |
| 27 | 43,356 | 0.72 | 0.63 | 0.86 |
| 28 | 42,683 | 17.65 | 2.89 | 0.16 |
| 29 | 41,603 | 1.39 | 1.04 | 0.74 |
| 30 | 43,146 | 3.1 | 0.94 | 0.3 |
| Inclusion body myositis |  |  |  |  |
| 1 | 36,682 | 14.2 | 4.49 | 0.31 |
| 2 | 44,434 | 2.55 | 2.1 | 0.82 |
| 3 | 40,855 | 1.02 | 0.92 | 0.9 |
| 4 | 39,294 | 1.1 | 0.96 | 0.87 |
| 5 | 41,262 | 1.4 | 0.84 | 0.6 |
| 6 | 41,318 | 1.37 | 1.28 | 0.93 |
| 7 | 40,381 | 4.42 | 2.1 | 0.47 |
| 8 | 40,445 | 32.48 | 20.59 | 0.63 |
| 9 | 43,689 | 1.45 | 0.74 | 0.51 |
| 10 | 42,617 | 1.24 | 1.03 | 0.83 |
| 11 | 43,793 | 1.79 | 1.44 | 0.8 |
| 12 | 41,149 | 1.93 | 0.93 | 0.48 |
| 13 | 23,932 | 74.88 | 40.69 | 0.54 |
| 14 | 39,235 | 2.77 | 0.94 | 0.33 |
| 15 | 38,347 | 15.29 | 1.28 | 0.08 |
| 16 | 43,650 | 2.18 | 1.71 | 0.78 |
| 17 | 40,701 | 70.24 | 3.06 | 0.04 |
| 18 | 43,136 | 2.44 | 1.8 | 0.73 |
| 19 | 40,406 | 25.67 | 3.51 | 0.13 |
| 20 | 39,959 | 27.46 | 4.56 | 0.16 |
| 21 | 39,376 | 0.97 | 0.42 | 0.43 |
| 22 | 41,406 | 1.03 | 0.65 | 0.63 |
| 23 | 42,919 | 0.8 | 0.63 | 0.78 |
| 24 | 37,526 | 1.65 | 0.74 | 0.44 |
| 25 | 39,808 | 2.25 | 0.83 | 0.36 |
| 26 | 42,336 | 1.17 | 0.74 | 0.63 |
| 27 | 39,721 | 1.29 | 0.63 | 0.48 |
| 28 | 41,927 | 1.06 | 0.72 | 0.67 |
| 29 | 43,323 | 2.79 | 1.19 | 0.42 |
| 30 | 40,763 | 2.1 | 0.99 | 0.47 |
| Duchenne muscular dystrophy | |  |  |  |
| 1 | 38,325 | 52.7 | 1.89 | 0.03 |
| 2 | 36,965 | 6.94 | 3.36 | 0.48 |
| 3 | 35,981 | 1.25 | 0.73 | 0.58 |
| 4 | 36,702 | 6.72 | 1.36 | 0.2 |
| 5 | 35,317 | 2.28 | 0.89 | 0.39 |
| 6 | 38,782 | 84.58 | 29.57 | 0.34 |
| 7 | 36,872 | 4.03 | 1.02 | 0.25 |
| 8 | 37,415 | 2.26 | 0.79 | 0.34 |
| 9 | 37,750 | 18.58 | 2.58 | 0.13 |
| 10 | 35,935 | 1.19 | 0.6 | 0.5 |
| 11 | 37,233 | 57.99 | 10.08 | 0.17 |
| 12 | 37,507 | 18.79 | 1.01 | 0.05 |
| 13 | 36,372 | 1.62 | 0.77 | 0.47 |
| 14 | 37,445 | 3.43 | 1.04 | 0.3 |
| 15 | 37,069 | 0.92 | 0.57 | 0.61 |
| 16 | 37,555 | 4.36 | 1.07 | 0.24 |
| 17 | 40,544 | 1.4 | 0.75 | 0.53 |
| 18 | 37,424 | 3.51 | 1.78 | 0.5 |
| 19 | 37,045 | 2.39 | 1.21 | 0.5 |
| 20 | 40,593 | 1.08 | 0.58 | 0.53 |
| 21 | 40,336 | 0.97 | 0.67 | 0.69 |
| 22 | 35,604 | 3.5 | 1.51 | 0.43 |
| 23 | 37,069 | 1.88 | 1.21 | 0.64 |
| 24 | 36,875 | 3.6 | 1.26 | 0.35 |
| 25 | 40,914 | 0.92 | 0.66 | 0.71 |
| 26 | 36,945 | 1.06 | 0.9 | 0.84 |
| 27 | 37,264 | 85.78 | 36.37 | 0.42 |
| 28 | 36,210 | 1.31 | 0.9 | 0.68 |
| 29 | 40,645 | 3.77 | 1.05 | 0.27 |
| 30 | 34,573 | 1.49 | 0.8 | 0.53 |
| Healthy control |  |  |  |  |
| 1 | 38,476 | 2.65 | 1.11 | 0.41 |
| 2 | 40,273 | 1.17 | 0.83 | 0.7 |
| 3 | 38,707 | 1.84 | 0.71 | 0.38 |
| 4 | 41,423 | 0.71 | 0.47 | 0.66 |
| 5 | 40,951 | 1.14 | 0.79 | 0.69 |
| 6 | 38,918 | 3.37 | 1.79 | 0.53 |
| 7 | 40,613 | 0.81 | 0.5 | 0.61 |
| 8 | 9,179 | 1.68 | 0.69 | 0.41 |
| 9 | 41,403 | 0.78 | 0.71 | 0.91 |
| 10 | 12,919 | 0.89 | 0.4 | 0.44 |
| 11 | 41,498 | 1.11 | 0.63 | 0.56 |
| 12 | 41,508 | 1.43 | 0.69 | 0.48 |
| 13 | 41,805 | 1.76 | 0.82 | 0.46 |
| 14 | 43,032 | 0.76 | 0.67 | 0.88 |
| 15 | 41,938 | 2.69 | 1.05 | 0.39 |
| 16 | 38,216 | 3.67 | 0.77 | 0.2 |
| 17 | 25,233 | 1.32 | 0.82 | 0.62 |
| 18 | 37,700 | 21.94 | 2.18 | 0.09 |
| 19 | 41,797 | 42.72 | 22.67 | 0.53 |
| 20 | 41,216 | 0.78 | 0.55 | 0.7 |
| 21 | 35,972 | 1.67 | 1 | 0.59 |
| 22 | 40,416 | 0.88 | 0.65 | 0.73 |
| 23 | 22,213 | 4.14 | 2.43 | 0.58 |
| 24 | 18,868 | 0.73 | 0.45 | 0.61 |
| 25 | 40,326 | 0.9 | 0.84 | 0.93 |
| 26 | 39,933 | 0.91 | 0.8 | 0.87 |
| 27 | 41,058 | 12.31 | 2.11 | 0.17 |
| 28 | 40,754 | 1.19 | 0.91 | 0.76 |
| 29 | 41,188 | 1.42 | 0.71 | 0.5 |
| 30 | 22,595 | 1.86 | 1.17 | 0.62 |

Supplementary data 3

Anti-Kv1.4 antibody index

| Kv1.4-transfected cells | Cell number | Proportion of PE positive 293F cells | Proportion of PE positive titin-transfected cells | Antibody index |
| --- | --- | --- | --- | --- |
| MG with Myositis and/or Myocarditis |  |  |  |  |
| 1 | 42,320 | 1.03 | 1.41 | 1.36 |
| 2 | 42,815 | 0.63 | 0.96 | 1.52 |
| 3 | 35,019 | 0.57 | 0.67 | 1.17 |
| 4 | 33,284 | 1.66 | 0.04 | 0.02 |
| 5 | 45,337 | 11.06 | 13.98 | 1.26 |
| 6 | 45,597 | 43.85 | 51.47 | 1.17 |
| 7 | 34,790 | 1.61 | 0.72 | 0.44 |
| 8 | 45,688 | 0.79 | 1.32 | 1.67 |
| 9 | 39,259 | 3.49 | 0.04 | 0.01 |
| 10 | 45,446 | 35.37 | 38.63 | 1.09 |
| 11 | 30,849 | 1.17 | 0.68 | 0.58 |
| 12 | 27,610 | 2.3 | 0.83 | 0.36 |
| 13 | 42,470 | 1.6 | 0.13 | 0.08 |
| 14 | 45,637 | 13.26 | 5.27 | 0.39 |
| 15 | 45,962 | 0.8 | 1.12 | 1.4 |
| 16 | 44,409 | 79.11 | 96.48 | 1.21 |
| 17 | 37,706 | 0.88 | 1.61 | 1.82 |
| 18 | 24,742 | 1.47 | 0.9 | 0.61 |
| 19 | 42,219 | 1.25 | 0.49 | 0.39 |
| 20 | 37,815 | 1.65 | 3.14 | 1.9 |
| 21 | 23,999 | 99.75 | 98.67 | 0.98 |
| 22 | 43,963 | 80.16 | 18.41 | 0.22 |
| 23 | 44,473 | 1.73 | 1.63 | 0.94 |
| 24 | 45,393 | 3.52 | 7.04 | 2 |
| 25 | 39,602 | 22.56 | 39.53 | 1.75 |
| 26 | 39,365 | 5.86 | 3.33 | 0.56 |
| 27 | 40,202 | 27.72 | 87.83 | 3.16 |
| 28 | 43,690 | 1.58 | 1.13 | 0.71 |
| 29 | 37,736 | 84.49 | 97.62 | 1.15 |
| 30 | 30,302 | 1.86 | 0.16 | 0.08 |
| Early-onset MG |  |  |  |  |
| 1 | 45,387 | 1.42 | 1.08 | 0.76 |
| 2 | 36,701 | 6.05 | 0.68 | 0.11 |
| 3 | 45,449 | 31.41 | 1.77 | 0.05 |
| 4 | 44,294 | 4.04 | 1.46 | 0.36 |
| 5 | 23,125 | 1.93 | 0.97 | 0.5 |
| 6 | 44,430 | 8.22 | 2.09 | 0.25 |
| 7 | 22,158 | 1.28 | 0.66 | 0.51 |
| 8 | 21,551 | 5.36 | 2.17 | 0.4 |
| 9 | 22,754 | 1.1 | 0.55 | 0.5 |
| 10 | 20,022 | 1.71 | 0.86 | 0.5 |
| 11 | 22,311 | 0.82 | 0.44 | 0.53 |
| 12 | 41,685 | 1.76 | 0.92 | 0.52 |
| 13 | 21,802 | 0.77 | 0.4 | 0.51 |
| 14 | 44,444 | 48.56 | 10.71 | 0.22 |
| 15 | 45,086 | 3.26 | 0.78 | 0.23 |
| 16 | 43,121 | 3.46 | 1.44 | 0.41 |
| 17 | 23,446 | 1.31 | 0.86 | 0.65 |
| 18 | 23,621 | 0.44 | 0.53 | 1.2 |
| 19 | 44,029 | 36.72 | 3.88 | 0.1 |
| 20 | 22,578 | 1.77 | 0.89 | 0.5 |
| 21 | 21,664 | 4.46 | 2.52 | 0.56 |
| 22 | 46,244 | 1.3 | 0.63 | 0.48 |
| 23 | 22,624 | 75.81 | 42.17 | 0.55 |
| 24 | 44,937 | 5.9 | 1.57 | 0.26 |
| 25 | 22,838 | 6.32 | 3.04 | 0.48 |
| 26 | 23,438 | 2.93 | 2.06 | 0.7 |
| 27 | 31,768 | 14.11 | 5.73 | 0.4 |
| 28 | 23,213 | 10.42 | 4.71 | 0.45 |
| 29 | 45,356 | 1.16 | 0.59 | 0.5 |
| 30 | 45,221 | 0.53 | 0.59 | 1.11 |
| Late-onset MG |  |  |  |  |
| 1 | 21,541 | 0.57 | 1.01 | 1.77 |
| 2 | 19,961 | 4.59 | 1.49 | 0.32 |
| 3 | 43,032 | 2.18 | 31.8 | 14.59 |
| 4 | 25,875 | 28.64 | 9.06 | 0.31 |
| 5 | 16,759 | 1.3 | 0.59 | 0.45 |
| 6 | 19,265 | 2.12 | 25.3 | 11.93 |
| 7 | 41,560 | 3.45 | 1.1 | 0.31 |
| 8 | 45,625 | 3.14 | 2.51 | 0.8 |
| 9 | 20,997 | 1.67 | 1.99 | 1.19 |
| 10 | 18,405 | 4.08 | 1.57 | 0.38 |
| 11 | 44,792 | 38.33 | 7.99 | 0.2 |
| 12 | 20,548 | 0.98 | 1.99 | 2.03 |
| 13 | 44,642 | 14.68 | 2.55 | 0.17 |
| 14 | 17,662 | 0.66 | 0.9 | 1.36 |
| 15 | 44,011 | 28.98 | 8.43 | 0.29 |
| 16 | 43,918 | 1.72 | 1.56 | 0.91 |
| 17 | 44,829 | 0.63 | 1.17 | 1.86 |
| 18 | 21,866 | 4.39 | 1.61 | 0.36 |
| 19 | 19,603 | 5.64 | 4.85 | 0.86 |
| 20 | 20,299 | 0.9 | 2 | 1.11 |
| 21 | 19,287 | 10.68 | 2.74 | 0.25 |
| 22 | 24,027 | 0.85 | 1.82 | 2.14 |
| 23 | 43,486 | 1.41 | 0.64 | 0.45 |
| 24 | 42,272 | 1.22 | 0.48 | 0.39 |
| 25 | 22,282 | 0.84 | 0.43 | 0.51 |
| 26 | 23,085 | 0.71 | 1.45 | 2.04 |
| 27 | 42,483 | 97.06 | 60.76 | 0.62 |
| 28 | 21,680 | 1.92 | 0.79 | 0.41 |
| 29 | 25,527 | 1.06 | 3.02 | 2.85 |
| 30 | 42,282 | 2.14 | 0.58 | 0.27 |
| Thymoma-associated MG |  |  |  |  |
| 1 | 20,300 | 2.35 | 1 | 0.42 |
| 2 | 20,741 | 2.94 | 0.97 | 0.32 |
| 3 | 44,440 | 2.12 | 1.23 | 0.58 |
| 4 | 44,043 | 2.56 | 1.15 | 0.44 |
| 5 | 44,796 | 0.75 | 0.95 | 1.26 |
| 6 | 40,477 | 0.9 | 0.43 | 0.47 |
| 7 | 41,707 | 0.87 | 0.41 | 0.47 |
| 8 | 39,747 | 2.95 | 0.63 | 0.21 |
| 9 | 39,971 | 36.28 | 2.96 | 0.08 |
| 10 | 39,093 | 1.7 | 0.38 | 0.22 |
| 11 | 39,127 | 17.65 | 2.09 | 0.11 |
| 12 | 44,724 | 0.68 | 0.89 | 1.3 |
| 13 | 44,925 | 0.56 | 1.1 | 1.96 |
| 14 | 45,110 | 0.56 | 0.59 | 1.05 |
| 15 | 45,197 | 0.61 | 0.82 | 1.34 |
| 16 | 37,224 | 2.61 | 0.56 | 0.21 |
| 17 | 45,032 | 0.53 | 0.99 | 1.86 |
| 18 | 43,762 | 0.64 | 0.89 | 1.39 |
| 19 | 44,826 | 0.54 | 0.87 | 1.61 |
| 20 | 44,373 | 14.14 | 5.72 | 0.4 |
| 21 | 40,008 | 1.04 | 0.36 | 0.34 |
| 22 | 44,522 | 0.57 | 0.72 | 1.26 |
| 23 | 41,144 | 0.59 | 0.84 | 1.42 |
| 24 | 38,618 | 9.63 | 1.05 | 0.1 |
| 25 | 44,781 | 1 | 1.51 | 1.51 |
| 26 | 39,625 | 41.4 | 5.14 | 0.12 |
| 27 | 44,316 | 81.41 | 65.93 | 0.8 |
| 28 | 43,407 | 10.12 | 1.07 | 0.1 |
| 29 | 24,702 | 0.49 | 0.55 | 1.12 |
| 30 | 39,154 | 1.47 | 0.32 | 0.21 |
| Anti-signal recognition particle myopathy |  |  |  |  |
| 1 | 41,323 | 41.55 | 5.11 | 0.12 |
| 2 | 40,087 | 73.3 | 18.55 | 0.25 |
| 3 | 40,800 | 25.08 | 2.66 | 0.1 |
| 4 | 44,618 | 3.1 | 2.26 | 0.72 |
| 5 | 40,374 | 28.8 | 3.04 | 0.1 |
| 6 | 41,521 | 1.85 | 2.22 | 1.2 |
| 7 | 42,350 | 1.09 | 1.68 | 1.54 |
| 8 | 41,316 | 1.68 | 2.56 | 1.52 |
| 9 | 40,555 | 13.56 | 6.08 | 0.44 |
| 10 | 40,299 | 1.8 | 1.13 | 0.63 |
| 11 | 36,603 | 33.34 | 2.9 | 0.08 |
| 12 | 35,294 | 10.64 | 1.42 | 0.13 |
| 13 | 41,538 | 3.96 | 1 | 0.25 |
| 14 | 34,709 | 5.32 | 1.18 | 0.22 |
| 15 | 34,680 | 3.11 | 1.11 | 0.35 |
| 16 | 34,714 | 8.2 | 1.25 | 0.15 |
| 17 | 32,534 | 66.54 | 33.99 | 0.51 |
| 18 | 33,567 | 12.33 | 1.72 | 0.13 |
| 19 | 35,124 | 3.91 | 1.01 | 0.25 |
| 20 | 35,548 | 31.14 | 3.11 | 0.09 |
| 21 | 34,348 | 4.5 | 0.92 | 0.2 |
| 22 | 35,676 | 12.39 | 1.22 | 0.09 |
| 23 | 34,455 | 3.04 | 0.96 | 0.31 |
| 24 | 39,123 | 25.65 | 3.04 | 0.11 |
| 25 | 35,236 | 20.87 | 2.24 | 0.1 |
| 26 | 37,001 | 4.76 | 0.89 | 0.18 |
| 27 | 41,660 | 1.17 | 0.96 | 0.82 |
| 28 | 36,261 | 5.92 | 1.15 | 0.19 |
| 29 | 34,994 | 2.93 | 0.99 | 0.33 |
| 30 | 36,123 | 1.9 | 0.65 | 0.34 |
| Inclusion body myositis |  |  |  |  |
| 1 | 38,459 | 9 | 1.05 | 0.11 |
| 2 | 35,758 | 7.04 | 6.85 | 0.97 |
| 3 | 41,262 | 1.02 | 0.95 | 0.93 |
| 4 | 35,606 | 32.59 | 22.32 | 0.68 |
| 5 | 12,708 | 4.06 | 0.71 | 0.17 |
| 6 | 41,816 | 0.76 | 0.61 | 0.8 |
| 7 | 13,411 | 7.35 | 1.51 | 0.2 |
| 8 | 44,655 | 42.75 | 14.74 | 0.34 |
| 9 | 44,869 | 1.07 | 0.69 | 0.64 |
| 10 | 14,682 | 0.76 | 0.41 | 0.53 |
| 11 | 45,212 | 0.73 | 0.6 | 0.82 |
| 12 | 44,543 | 1.46 | 0.73 | 0.5 |
| 13 | 42,560 | 2.43 | 1.76 | 0.72 |
| 14 | 41,617 | 0.9 | 0.55 | 0.61 |
| 15 | 36,118 | 66.17 | 59.74 | 0.9 |
| 16 | 44,497 | 1.25 | 0.82 | 0.65 |
| 17 | 28,447 | 57.88 | 12.55 | 0.21 |
| 18 | 44,576 | 1.21 | 0.93 | 0.76 |
| 19 | 19,409 | 6.03 | 0.08 | 0.01 |
| 20 | 44,472 | 2.59 | 1.06 | 0.4 |
| 21 | 36,139 | 1.35 | 0.92 | 0.68 |
| 22 | 44,491 | 0.76 | 0.64 | 0.84 |
| 23 | 44,055 | 0.61 | 0.74 | 1.21 |
| 24 | 36,965 | 0.62 | 1.1 | 1.77 |
| 25 | 38,308 | 2.57 | 1.74 | 0.67 |
| 26 | 44,600 | 0.79 | 0.55 | 0.69 |
| 27 | 39,200 | 0.96 | 1.15 | 1.19 |
| 28 | 39,707 | 0.93 | 1.45 | 1.55 |
| 29 | 44,620 | 3.12 | 1.03 | 0.33 |
| 30 | 44,512 | 2.43 | 0.92 | 0.37 |
| Duchenne muscular dystrophy | |  |  |  |
| 1 | 35,674 | 73 | 17.58 | 0.24 |
| 2 | 45,558 | 18.65 | 1.6 | 0.08 |
| 3 | 40,794 | 1.17 | 0.63 | 0.53 |
| 4 | 36,534 | 13.34 | 2.36 | 0.17 |
| 5 | 35,775 | 2.33 | 1.34 | 0.57 |
| 6 | 39,678 | 99.64 | 89.32 | 0.89 |
| 7 | 36,951 | 2.62 | 1.08 | 0.41 |
| 8 | 36,456 | 4.85 | 1.23 | 0.25 |
| 9 | 37,157 | 7.3 | 1.33 | 0.18 |
| 10 | 40,897 | 3.18 | 0.89 | 0.27 |
| 11 | 35,574 | 64.31 | 11.46 | 0.17 |
| 12 | 39,579 | 4.9 | 1.2 | 0.24 |
| 13 | 41,174 | 0.79 | 0.57 | 0.72 |
| 14 | 38,270 | 2.14 | 1.16 | 0.54 |
| 15 | 34,559 | 0.7 | 0.78 | 1.11 |
| 16 | 35,286 | 4.03 | 1.58 | 0.39 |
| 17 | 35,856 | 25.66 | 3.8 | 0.14 |
| 18 | 364,52 | 23.11 | 4.08 | 0.17 |
| 19 | 35,243 | 70.65 | 14.79 | 0.2 |
| 20 | 34,305 | 9.79 | 1.94 | 0.19 |
| 21 | 33,511 | 0.97 | 1.02 | 1.05 |
| 22 | 38,902 | 1.62 | 1 | 0.61 |
| 23 | 39,378 | 0.82 | 0.56 | 0.68 |
| 24 | 42,278 | 17.83 | 3.79 | 0.21 |
| 25 | 39,146 | 0.95 | 0.89 | 0.93 |
| 26 | 39,032 | 1.54 | 0.99 | 0.64 |
| 27 | 36,626 | 6.23 | 3.12 | 0.5 |
| 28 | 40,153 | 1.49 | 0.63 | 0.42 |
| 29 | 39,739 | 1.46 | 1.21 | 0.82 |
| 30 | 39,299 | 1.62 | 0.66 | 0.4 |
| Healthy control |  |  |  |  |
| 1 | 41,474 | 1.05 | 0.27 | 0.25 |
| 2 | 35,481 | 2.18 | 1.57 | 0.72 |
| 3 | 41,529 | 1.07 | 0.23 | 0.21 |
| 4 | 39,463 | 1.76 | 1.03 | 0.58 |
| 5 | 35,815 | 1.29 | 1.13 | 0.87 |
| 6 | 32,907 | 1.67 | 1.17 | 0.7 |
| 7 | 35,772 | 0.63 | 0.35 | 0.55 |
| 8 | 38,964 | 0.01 | 0.01 | 1 |
| 9 | 41,632 | 0.5 | 0.22 | 0.44 |
| 10 | 42,871 | 0.59 | 0.26 | 0.44 |
| 11 | 40,578 | 0.67 | 0.54 | 0.8 |
| 12 | 39,450 | 0.79 | 0.42 | 0.53 |
| 13 | 41,325 | 1.04 | 0.89 | 0.85 |
| 14 | 41,922 | 0.72 | 0.57 | 0.79 |
| 15 | 41,913 | 1.92 | 0.61 | 0.31 |
| 16 | 41,121 | 1.72 | 0.79 | 0.45 |
| 17 | 41,150 | 3.41 | 0.81 | 0.23 |
| 18 | 39,966 | 1.82 | 0.53 | 0.29 |
| 19 | 41,603 | 50.86 | 7.95 | 0.15 |
| 20 | 24,275 | 1.07 | 0.61 | 0.57 |
| 21 | 40,567 | 2.04 | 0.95 | 0.46 |
| 22 | 40,043 | 0.9 | 0.43 | 0.47 |
| 23 | 40,051 | 0.7 | 0.41 | 0.58 |
| 24 | 31,090 | 1.12 | 0.76 | 0.67 |
| 25 | 40,535 | 1.03 | 0.47 | 0.45 |
| 26 | 39,633 | 0.7 | 0.51 | 0.72 |
| 27 | 40,008 | 1.22 | 0.67 | 0.54 |
| 28 | 40,059 | 1.21 | 0.65 | 0.53 |
| 29 | 38,062 | 6.86 | 0.55 | 0.08 |
| 30 | 39,895 | 0.81 | 0.5 | 0.61 |

Supplementary data 4

Anti-ryanodine receptor (RyR) antibody index

| RyR-transfected cells | Cell number | Proportion of PE positive 293F cells | Proportion of PE positive titin-transfected cells | Antibody index |
| --- | --- | --- | --- | --- |
| MG with Myositis and/or Myocarditis |  |  |  |  |
| 1 | 38,363 | 38.21 | 52.22 | 1.36 |
| 2 | 41,571 | 0.84 | 1.32 | 1.57 |
| 3 | 29,683 | 98.23 | 85.22 | 0.87 |
| 4 | 38,642 | 61.4 | 67.92 | 1.1 |
| 5 | 39,375 | 67.9 | 86.04 | 1.26 |
| 6 | 39,076 | 66.76 | 80.98 | 1.21 |
| 7 | 38,966 | 1.14 | 0.9 | 0.78 |
| 8 | 39,236 | 12.09 | 27.58 | 2.28 |
| 9 | 41,571 | 0.84 | 0.31 | 0.37 |
| 10 | 39,078 | 76.43 | 93.59 | 1.22 |
| 11 | 39,267 | 27.76 | 20.62 | 0.74 |
| 12 | 38,533 | 6.96 | 11.67 | 1.67 |
| 13 | 40,701 | 0.49 | 0.4 | 0.81 |
| 14 | 38,430 | 65.36 | 86.11 | 1.31 |
| 15 | 39,368 | 4.07 | 2.11 | 0.52 |
| 16 | 39,357 | 93.02 | 97.59 | 1.04 |
| 17 | 28,454 | 2.15 | 2.2 | 1.02 |
| 18 | 41,144 | 0.52 | 0.56 | 1.07 |
| 19 | 39,508 | 4.04 | 7.17 | 1.77 |
| 20 | 39,895 | 35.04 | 52.46 | 1.49 |
| 21 | 36,449 | 40.62 | 64.68 | 1.59 |
| 22 | 38,061 | 16.78 | 1.93 | 0.11 |
| 23 | 36,481 | 4.56 | 4.88 | 1.07 |
| 24 | 38,683 | 53.12 | 87.38 | 1.64 |
| 25 | 31,663 | 23.98 | 31.18 | 1.3 |
| 26 | 31,785 | 3.74 | 10.11 | 2.7 |
| 27 | 27,909 | 1.09 | 1.57 | 1.44 |
| 28 | 38,410 | 31.72 | 30.17 | 0.95 |
| 29 | 32,864 | 5.5 | 10.25 | 1.86 |
| 30 | 40,832 | 0.63 | 0.82 | 1.3 |
| Early-onset MG |  |  |  |  |
| 1 | 37,008 | 1.11 | 1.61 | 1.45 |
| 2 | 34,606 | 20.23 | 9.02 | 0.44 |
| 3 | 36,394 | 1.85 | 2.95 | 1.59 |
| 4 | 38,742 | 0.92 | 0.57 | 0.61 |
| 5 | 16,141 | 11.17 | 5.85 | 0.52 |
| 6 | 38,647 | 1.53 | 1.8 | 1.17 |
| 7 | 38,550 | 1.65 | 1.29 | 0.78 |
| 8 | 13,614 | 96.56 | 97.04 | 1 |
| 9 | 39,817 | 65.44 | 74.42 | 1.13 |
| 10 | 13,641 | 0.82 | 0.51 | 0.62 |
| 11 | 43,025 | 1.22 | 0.97 | 0.79 |
| 12 | 42,510 | 0.77 | 1.25 | 1.62 |
| 13 | 27,777 | 0.67 | 0.57 | 0.85 |
| 14 | 18,220 | 1.81 | 0.65 | 0.35 |
| 15 | 19,719 | 32.15 | 18.8 | 0.58 |
| 16 | 22,305 | 9.43 | 4.36 | 0.46 |
| 17 | 40,120 | 14.48 | 27.59 | 1.9 |
| 18 | 23,253 | 0.52 | 0.31 | 0.59 |
| 19 | 26,717 | 12.16 | 3.09 | 0.25 |
| 20 | 41,374 | 10.52 | 2.94 | 0.27 |
| 21 | 38,849 | 96.32 | 99.58 | 1.03 |
| 22 | 35,376 | 2.03 | 2.24 | 1.1 |
| 23 | 21,681 | 87.49 | 69.97 | 0.79 |
| 24 | 41,828 | 16.92 | 3.68 | 0.21 |
| 25 | 19,117 | 41.16 | 19.95 | 0.48 |
| 26 | 41,601 | 5.02 | 16.76 | 3.33 |
| 27 | 13,213 | 0.53 | 0.19 | 0.35 |
| 28 | 24,799 | 97.56 | 96.93 | 0.99 |
| 29 | 25,254 | 0.96 | 0.4 | 0.41 |
| 30 | 23,398 | 0.8 | 1.14 | 1.42 |
| Late-onset MG |  |  |  |  |
| 1 | 33,757 | 14.12 | 15.57 | 1.1 |
| 2 | 35,408 | 3.14 | 2.28 | 0.72 |
| 3 | 35,071 | 8.99 | 7.17 | 0.79 |
| 4 | 33,031 | 2.32 | 2.4 | 1.03 |
| 5 | 34,850 | 2.25 | 3.41 | 1.51 |
| 6 | 34,448 | 3.11 | 4.99 | 1.6 |
| 7 | 34,050 | 19.72 | 30.19 | 1.53 |
| 8 | 30,777 | 2.34 | 1.52 | 0.64 |
| 9 | 32,957 | 5.44 | 3.03 | 0.55 |
| 10 | 34,997 | 6.15 | 7.46 | 1.21 |
| 11 | 33,981 | 15.81 | 25.37 | 1.6 |
| 12 | 33,340 | 2.12 | 2.85 | 1.34 |
| 13 | 36,661 | 2.53 | 2.6 | 1.02 |
| 14 | 33,425 | 6.94 | 9.43 | 1.35 |
| 15 | 19,574 | 2.87 | 2.5 | 0.87 |
| 16 | 33,858 | 6.88 | 3.47 | 0.5 |
| 17 | 35,028 | 1.4 | 1.07 | 0.76 |
| 18 | 34,120 | 21.16 | 11.4 | 0.53 |
| 19 | 33,160 | 5.46 | 8.55 | 1.56 |
| 20 | 33,270 | 3.98 | 2.72 | 0.68 |
| 21 | 13,516 | 1.19 | 0.36 | 0.3 |
| 22 | 34,008 | 1.46 | 1.7 | 1.16 |
| 23 | 35,881 | 0.96 | 0.71 | 0.73 |
| 24 | 33,401 | 2.78 | 1.96 | 0.7 |
| 25 | 33,675 | 4.39 | 2.86 | 0.65 |
| 26 | 33,527 | 5.77 | 3.87 | 0.67 |
| 27 | 18,978 | 20.42 | 28.1 | 1.37 |
| 28 | 20,919 | 2.81 | 2.27 | 0.8 |
| 29 | 32,899 | 9.76 | 6.95 | 0.71 |
| 30 | 34,801 | 3.32 | 2.22 | 0.66 |
| Thymoma-associated MG |  |  |  |  |
| 1 | 38,431 | 13.57 | 24.52 | 1.8 |
| 2 | 35,349 | 3.04 | 1.87 | 0.61 |
| 3 | 26,054 | 0.85 | 0.32 | 0.37 |
| 4 | 21,039 | 3.24 | 1.15 | 0.35 |
| 5 | 33,366 | 2.74 | 4.59 | 1.67 |
| 6 | 39,684 | 1.65 | 4.13 | 2.5 |
| 7 | 35,951 | 0.89 | 0.93 | 1.04 |
| 8 | 36,234 | 76.12 | 90.89 | 1.19 |
| 9 | 40,692 | 29.34 | 7.71 | 0.26 |
| 10 | 34,916 | 3.71 | 5.96 | 1.6 |
| 11 | 36,871 | 5.11 | 4.52 | 0.88 |
| 12 | 24,636 | 0.97 | 1.32 | 1.36 |
| 13 | 40,151 | 1.18 | 1.13 | 0.95 |
| 14 | 39,468 | 1.03 | 1.32 | 1.28 |
| 15 | 39,681 | 2.19 | 2.02 | 0.92 |
| 16 | 36,980 | 1.74 | 1.13 | 0.64 |
| 17 | 40,390 | 1.67 | 1.96 | 1.17 |
| 18 | 40,433 | 0.7 | 0.31 | 0.44 |
| 19 | 33,465 | 1.45 | 1.26 | 0.86 |
| 20 | 41,002 | 3.67 | 2.02 | 0.55 |
| 21 | 40,148 | 1.75 | 2 | 1.14 |
| 22 | 40,251 | 1.77 | 1.88 | 1.06 |
| 23 | 37,863 | 1.66 | 1.39 | 0.83 |
| 24 | 40,953 | 35.54 | 24.98 | 0.7 |
| 25 | 38,725 | 5.9 | 5.5 | 0.93 |
| 26 | 40,002 | 0.79 | 0.82 | 1.03 |
| 27 | 37,601 | 95.19 | 97.44 | 1.02 |
| 28 | 22,695 | 0.94 | 0.65 | 0.69 |
| 29 | 36,722 | 1.25 | 1.09 | 0.87 |
| 30 | 35,032 | 1.79 | 1.65 | 0.92 |
| Anti-signal recognition particle myopathy |  |  |  |  |
| 1 | 30,325 | 2.65 | 3.58 | 1.35 |
| 2 | 31,447 | 45.11 | 46.84 | 1.03 |
| 3 | 31,088 | 1.26 | 1.76 | 1.39 |
| 4 | 31,475 | 9.86 | 15.88 | 1.61 |
| 5 | 32,383 | 7.29 | 12.62 | 1.73 |
| 6 | 32,372 | 29.08 | 34.86 | 1.19 |
| 7 | 31,993 | 4.81 | 5.03 | 1.04 |
| 8 | 30,986 | 2.61 | 3.5 | 1.34 |
| 9 | 31,487 | 98.96 | 99.2 | 1 |
| 10 | 31,453 | 4.48 | 0.93 | 0.2 |
| 11 | 30,701 | 14.21 | 12.12 | 0.85 |
| 12 | 31,964 | 41.4 | 52.39 | 1.26 |
| 13 | 34,304 | 4.79 | 8.1 | 1.69 |
| 14 | 32,314 | 20.19 | 40.22 | 1.99 |
| 15 | 31,655 | 56.71 | 67.92 | 1.19 |
| 16 | 31,075 | 40.29 | 42.86 | 1.06 |
| 17 | 34,560 | 99.52 | 99.88 | 1 |
| 18 | 32,787 | 30.34 | 64.52 | 2.12 |
| 19 | 32,014 | 34.59 | 46.85 | 1.35 |
| 20 | 35,804 | 9.93 | 20.28 | 2.04 |
| 21 | 32,748 | 20.01 | 39.15 | 1.95 |
| 22 | 32,141 | 19.48 | 19.86 | 1.01 |
| 23 | 32,241 | 13.07 | 23.88 | 1.82 |
| 24 | 32,523 | 10.19 | 29.58 | 2.9 |
| 25 | 32,737 | 28.6 | 47.1 | 1.64 |
| 26 | 34,052 | 14.76 | 21.34 | 1.44 |
| 27 | 32,162 | 22.26 | 48.07 | 2.15 |
| 28 | 33,276 | 32.3 | 47.21 | 1.46 |
| 29 | 32,549 | 6.83 | 12.66 | 1.85 |
| 30 | 34,279 | 62.35 | 88.54 | 1.42 |
| Inclusion body myositis |  |  |  |  |
| 1 | 36,357 | 67.96 | 51.67 | 0.76 |
| 2 | 34,529 | 20.72 | 6.75 | 0.32 |
| 3 | 29,864 | 80.86 | 85.11 | 1.05 |
| 4 | 32,890 | 4.24 | 15.01 | 3.54 |
| 5 | 33,348 | 65.61 | 79.74 | 1.21 |
| 6 | 28,831 | 11.77 | 16.64 | 1.41 |
| 7 | 23,156 | 57.28 | 54.03 | 0.94 |
| 8 | 29,833 | 94.31 | 93.68 | 0.99 |
| 9 | 34,338 | 1.25 | 1.7 | 1.36 |
| 10 | 33,085 | 3.96 | 4.73 | 1.19 |
| 11 | 33,810 | 1.86 | 4.44 | 2.39 |
| 12 | 35,017 | 5.53 | 2.51 | 0.45 |
| 13 | 17,701 | 91.76 | 88.45 | 0.96 |
| 14 | 37,705 | 3.14 | 18 | 5.73 |
| 15 | 35,019 | 35.51 | 6.3 | 0.17 |
| 16 | 35,394 | 20.22 | 4.5 | 0.22 |
| 17 | 20,161 | 98.03 | 94.04 | 0.95 |
| 18 | 34,764 | 15.64 | 4.06 | 0.25 |
| 19 | 23,089 | 20.03 | 8.67 | 0.43 |
| 20 | 37,606 | 8.28 | 15.71 | 1.89 |
| 21 | 38,805 | 2.53 | 9.43 | 3.72 |
| 22 | 36,296 | 0.9 | 1.31 | 1.45 |
| 23 | 35,294 | 1.96 | 1.58 | 0.8 |
| 24 | 40,566 | 9.36 | 46.3 | 4.95 |
| 25 | 25,511 | 7.33 | 7.64 | 1.04 |
| 26 | 27,622 | 7.94 | 7.82 | 0.98 |
| 27 | 33,017 | 6.35 | 6.88 | 1.08 |
| 28 | 27,566 | 14.7 | 11.37 | 0.77 |
| 29 | 31,847 | 69.16 | 17.06 | 0.24 |
| 30 | 20,177 | 19.34 | 14.38 | 0.74 |
| Duchenne muscular dystrophy | |  |  |  |
| 1 | 39,978 | 81.79 | 80.6 | 0.98 |
| 2 | 38,043 | 94.52 | 94.3 | 0.99 |
| 3 | 37,749 | 1.46 | 2.4 | 1.64 |
| 4 | 39,426 | 50.06 | 59.24 | 1.18 |
| 5 | 38,147 | 11.05 | 16.59 | 1.5 |
| 6 | 39,888 | 98.9 | 29.5 | 0.3 |
| 7 | 37,755 | 27.97 | 33.44 | 1.19 |
| 8 | 39,537 | 50.71 | 50.7 | 0.99 |
| 9 | 38,741 | 8.66 | 16.92 | 1.95 |
| 10 | 38,707 | 14.98 | 7.7 | 0.51 |
| 11 | 37,922 | 98.21 | 30.05 | 0.31 |
| 12 | 38,752 | 14.86 | 23.89 | 1.6 |
| 13 | 38,625 | 11.39 | 6.68 | 0.59 |
| 14 | 39,120 | 7.13 | 12.06 | 1.69 |
| 15 | 30,057 | 0.64 | 0.41 | 0.64 |
| 16 | 36,740 | 15.59 | 10 | 0.64 |
| 17 | 37,178 | 16.73 | 21.44 | 1.28 |
| 18 | 35,105 | 70.14 | 76.93 | 1.09 |
| 19 | 34,842 | 98.58 | 97.87 | 0.99 |
| 20 | 35,682 | 35.01 | 42.26 | 1.2 |
| 21 | 36,777 | 48.35 | 41.03 | 0.85 |
| 22 | 35,367 | 96.25 | 21.11 | 0.22 |
| 23 | 35,799 | 2.25 | 4.05 | 1.8 |
| 24 | 36,266 | 75.66 | 79.91 | 1.05 |
| 25 | 26,556 | 1.05 | 0.61 | 0.58 |
| 26 | 35,511 | 2.86 | 5.19 | 1.81 |
| 27 | 35,529 | 99.2 | 99.04 | 0.99 |
| 28 | 37,054 | 2.32 | 1.06 | 0.46 |
| 29 | 35,931 | 7.64 | 3.53 | 0.46 |
| 30 | 35,661 | 4.21 | 2.8 | 0.67 |
| Healthy control |  |  |  |  |
| 1 | 35,663 | 21.82 | 25.84 | 1.18 |
| 2 | 39,692 | 1.19 | 0.81 | 0.68 |
| 3 | 26,428 | 2.16 | 0.94 | 0.43 |
| 4 | 26,726 | 0.98 | 0.51 | 0.52 |
| 5 | 28,212 | 6.63 | 2.83 | 0.42 |
| 6 | 34,725 | 15.4 | 20.64 | 1.34 |
| 7 | 33,251 | 2.35 | 0.82 | 0.34 |
| 8 | 23,512 | 0.81 | 0.34 | 0.41 |
| 9 | 29,720 | 2.91 | 5.49 | 1.88 |
| 10 | 36,138 | 19.55 | 20.74 | 1.06 |
| 11 | 36,226 | 13.23 | 11.9 | 0.89 |
| 12 | 30,861 | 17.85 | 14.25 | 0.79 |
| 13 | 39,514 | 2.36 | 0.95 | 0.4 |
| 14 | 40,841 | 0.95 | 0.4 | 0.42 |
| 15 | 39,116 | 55.56 | 57.89 | 1.04 |
| 16 | 37,263 | 67.15 | 70.74 | 1.05 |
| 17 | 40,349 | 17.24 | 11.4 | 0.66 |
| 18 | 40,641 | 16.6 | 7.11 | 0.42 |
| 19 | 32,622 | 98.33 | 97.75 | 0.99 |
| 20 | 31,607 | 2.66 | 0.99 | 0.37 |
| 21 | 10,260 | 0.64 | 0.12 | 0.18 |
| 22 | 29,731 | 0.89 | 0.42 | 0.47 |
| 23 | 10,248 | 0.59 | 0.11 | 0.18 |
| 24 | 32,281 | 3.07 | 0.82 | 0.26 |
| 25 | 38,978 | 1.08 | 0.43 | 0.39 |
| 26 | 30,953 | 2.24 | 0.76 | 0.33 |
| 27 | 33,856 | 30.87 | 27.23 | 0.88 |
| 28 | 33,739 | 2.23 | 1.03 | 0.46 |
| 29 | 40,347 | 0.87 | 1.58 | 1.82 |
| 30 | 33,679 | 1.44 | 0.6 | 0.41 |
